# Ultrafast strong-field photoelectron emission from biased metal surfaces: exact solution to time-dependent Schrödinger Equation

**DOI:** 10.1038/srep19894

**Published:** 2016-01-28

**Authors:** Peng Zhang, Y. Y. Lau

**Affiliations:** 1 Department of Nuclear Engineering and Radiological Sciences, University of Michigan, Ann Arbor, Michigan 48109-2104, USA

## Abstract

Laser-driven ultrafast electron emission offers the possibility of manipulation and control of coherent electron motion in ultrashort spatiotemporal scales. Here, an analytical solution is constructed for the highly nonlinear electron emission from a dc biased metal surface illuminated by a single frequency laser, by solving the time-dependent Schrödinger equation exactly. The solution is valid for arbitrary combinations of dc electric field, laser electric field, laser frequency, metal work function and Fermi level. Various emission mechanisms, such as multiphoton absorption or emission, optical or dc field emission, are all included in this single formulation. The transition between different emission processes is analyzed in detail. The time-dependent emission current reveals that intense current modulation may be possible even with a low intensity laser, by merely increasing the applied dc bias. The results provide insights into the electron pulse generation and manipulation for many novel applications based on ultrafast laser-induced electron emission.

Ultrafast, laser-driven electron emission from metal nanostructures is of substantial current interest. The ultrashort coherent electron bunches produced^1^,^2^,^3^,^4^,^5^,^6^ are crucial to many areas, including free electron lasers (FELs)^7^, laser acceleration of relativistic electrons^8^, picosecond cathodoluminescence^9^, and femtosecond electron diffraction^10^,^11^. They would enable exciting technological developments such as four-dimensional (4D) time-resolved electron microscopy^12^, spectroscopy, and holography^3^,^13^,^14^,^15^, single-electron sources^16^, and carrier envelope phase detection^17^,^18^,^19^. The underlying emission mechanisms have been extensively studied, both theoretically^20^,^21^,^22^,^23^,^24^,^25^ and experimentally^1^,^2^,^3^,^4^,^14^,^17^. Bormann *et al.*^4^ experimentally demonstrated the transition from multiphoton absorption in relatively weak laser fields to optical field emission in strong laser fields. Yanagisawa *et al.*^13^,^14^ studied the electron energy distribution for laser induced electron emissions. The effects of local nonuniform fields^26^, dc bias^1^,^2^, and short pulse illumination^27^ have been individually studied. Perturbative theory^4^,^20^,^28^ was developed to model ultrafast electron emission, usually done with the strong field approximation. Floquet models were developed^20^,^29^, but only for electron emission from metals excited by a laser wave field. Numerical simulations^20^,^23^,^24^,^25^ are very powerful tools, but the physics for the interplay between various complicated electron emission processes is not always transparent.

Here, we present an analytical model to study electron emission from a metal surface, under the influence of both a dc electric field and laser field illumination. Our exact solution is applicable to electron emission for arbitrary values of laser field, laser frequency, dc electric field, and metal properties (i.e. work function and Fermi level). The different electron emission processes, such as multiphoton absorption, multiphoton emission, dc or optical field emission, single-photon induced over-barrier emission, and various combinations of them, are included in this single formulation. Detailed analyses on the effects of dc and laser field strength, laser frequency, barrier height, as well as time-dependent emission behaviors are presented. We found that applying a strong dc electric field for laser illuminated metal surface would open up more possible emission processes and introduce intense modulation to the time-dependent emitted current. Thus, this work provides insights not only to the susceptibility of electron emission mechanisms under the combination of ultrafast laser illumination and dc bias, but also to coherent quantum state manipulation of free electron populations^30^. As a check, our model recovers the known limiting cases of zero dc electric field^20^ and static field emission for zero laser field^31^,^32^.

## Results

### Analytical solution to time-dependent Schrödinger equation

**Figure 1:**
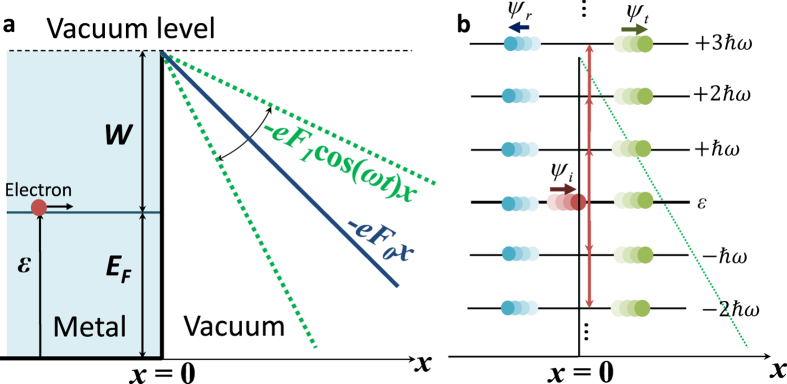
Energy diagram for strong field photoelectron emission. (**a**) The metal-vacuum interface under both laser illumination and dc bias. The metal has Fermi level *E*_*F*_ and work function *W*. The applied dc electric field is *F*_*0*_, the laser electric field is *F*_*1*_cos*ωt*. The initial electron energy is at *ε*. (**b**) The resulted ladder states for electrons with spacing of the photon energy 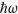 coupled to the initial electron energy at *ε*. The incident electron wave is *ψ*_*i*_, the transmitted and reflected electron waves are *ψ*_*t*_ and *ψ*_*r*_ respectively, both are superposition of plane waves with energies at the ladder states.

Consider electron emission from a metal-vacuum interface at *x* = 0 driven by a combination of a dc electric field *F*_*0*_ and a laser field *F*_*1*_cos(*ωt*) with a single carrier frequency *ω*, as shown in Fig. 1a. All electric fields are assumed to be perpendicular to the metal surface. The electrons in the metal would see a time varying potential barrier across the metal-vacuum interface,


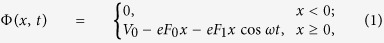


where 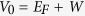, *E*_*F*_ and *W* are the Fermi energy and the work function of the metal respectively, and *e* is the electron charge. In eq. (1), the metal is approximated by jellium model, while the external fields are assumed cut off abruptly at the surface. The “sudden screening” of external fields may be justified, because the field penetration depth (i.e. skin depth) *δ*_*m*_* = *(2/*ωμ*_0_*σ*)^1/2^ of metal is typically much smaller than the laser wavelength (e. g. for gold, the conductivity *σ* = 4.1 × 10^7^ S/m, for 800 nm laser, *δ*_*m*_ = 4.06 nm). To calculate the emission probability, we solve the time-dependent Schrödinger equation,


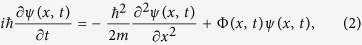


where *ψ*(*x*,*t*) is the complex electron wave function, *m* is the electron mass, and the potential Φ(*x*,*t*) is given in eq. (1). Following the coordinates transformation given by Truscott^33^,^34^, it is found that eq. (2) has an exact solution for *x* ≥ 0 (see Methods),


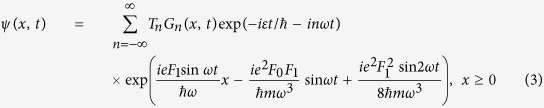


where *ε* is the initial energy of an incident electron, *T*_*n*_ is a constant representing the transmission amplitude, and *G*_*n*_(*x*, *t*) is given by,


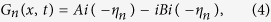


with 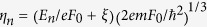, 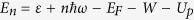, 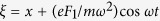, and 
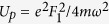
. It can be easily verified that eq. (3) is a solution of eq. (2) by direct substitution. Equation (4) represents an outgoing wave traveling to the right, with *Ai* and *Bi* being the Airy function of the first kind and second kind, respectively. It is clear that both *G*_*n*_(*t*) and the second exponential term in eq. (3) are time-periodic functions with period of 2*π*/*ω*, thus, eq. (3) represents the superposition of electron plane waves with energies of ladder states with spacing of the photon energy 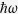 coupled to the initial energy at *ε*, as shown in Fig. 1b. The transfer of incident electrons at *ε* to sub-bands of 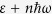 is made possible by processes such as multiphoton absorption (*n* > 0) or multiphoton emission (*n* < 0). Note that these ladder eigenstates are similar to Volkov states for a time-periodic potential^4^,^20^,^35^, whose eigenenergy 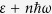 consists of a drift kinetic energy *E*_*n*_ and a ponderomotive energy *U*_*p*_ due to electron quiver motion, both of which are defined in the sentence following eq. (4).

For *x* < 0, the solution to eq. (2) may be written as,


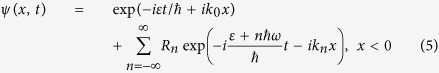


which is the superposition of an incident plane wave and a set of reflected waves with reflection amplitude *R*_*n*_, where 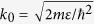, and 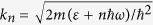. The reflected waves consist of an elastic reflected wave (*n* = 0) and waves with ladder eigenenergies representing multiple photon energy (*n* ≠ 0), as shown in Fig. 1b. Although the general solution in eq. (5) includes the reflected waves with *n* < 0, whose energies are below the initial electron energy *ε* (typically assumed to be the Fermi energy), it is verified that almost all the reflected current is through the initial energy level, i.e. *n* = 0. Whe*n* 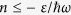, *k*_*n*_ becomes imaginary and the corresponding reflected wave becomes evanescent, resulting in zero reflected current through the channels with 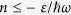. Note that in eq. (5) a plane wave is assumed for the incident electron, which turns out to be a good approximation to the bound-state electron wavefunction inside the metal solid, since its dimension in the longitudinal direction (perpendicular to the surface) is typically much larger than the electron wavelength. In fact, the laser field interacts only with the evanescent part of the electron states near the metal surface, which is found to be very similar to the bound-state wave function in the atomic case^29^.

The solutions in eqs (3) and (5) are matched at the metal-vacuum interface from the conditions that both *ψ* and *dψ*/*dx* are continuous at *x* = 0. By taking Fourier transform, we obtain, in nondimensional quantities, 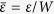, 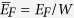, 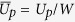, 
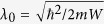
 (for typical work functions of metals 2 < *W* < 6 eV, 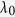 is of the order of 1 Angstrom), 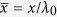, 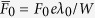, 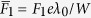, 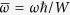, 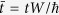,


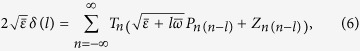


where *δ* is the Dirac delta function and,


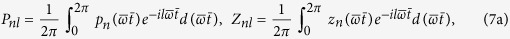



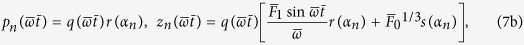


with 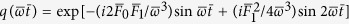, 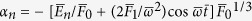, 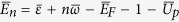, 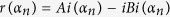, 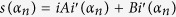, and a prime denotes derivative with respect to the argument. In eq. (7b), *p*_*n*_ and *z*_*n*_ represent the phase factor of the wavefunction in the *n*th state and of its spatial derivative at 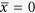, respectively. In eq. (7a), *P*_*nl*_ and *Z*_*nl*_ are the *l*th Fourier coefficients of *p*_*n*_ and *z*_*n*_ in eq. (7b), respectively. The summation rule in eq. (6) physically means the conservation of probability at the metal-vacuum interface, 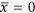. It is derived from the conditions that both the wave function and its first derivative be continuous there. The transmission amplitudes *T*_*n*_ is calculated from eq. (6). The normalized emission current density is defined as *w*(*ε*, *x*, *t*) ≡ *J*_*t*_(*ε*, *x*, *t*)/*J*_*i*_(*ε*), which is the ratio of the transmitted current density over the incident current density, calculated by using the probability current density 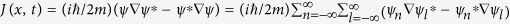. The emission current density is found in the normalized form as,


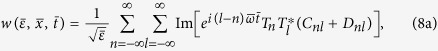



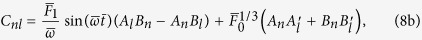



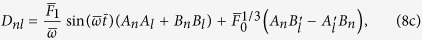


where 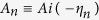, 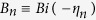, 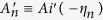, 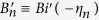, and 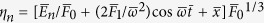. It is easy to show that the time averaged normalized emission current density is,


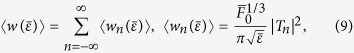


where 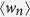 describes the emission current density through the *n*th channel, with emitted electrons of energy 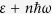, due to the *n*-photon contribution. Once the dc electric field *F*_*0*_, laser field *F*_*1*_, laser frequency *ω*, and metal properties (*E*_*F*_ and *W*) are specified, the time-dependent and time-averaged emission current density may be calculated from eqs (8) and (9) respectively, with *T*_*n*_ given by eq. (6), for any given initial electron energy *ε*. For simplicity, we take the energy of the initial electrons to be *ε* = *E*_*F*_, which is justified by the well-known fact that the probability of electron tunneling drops rapidly when electron energy decreases, with most of the electrons being emitted from sources near the Fermi level^20^,^23^,^31^,^32^,^36^. Numerical calculations have verified that the emission current drops exponentially as the initial electron energy decreases below the Fermi level.

### Main results and discussion

**Figure 2:**
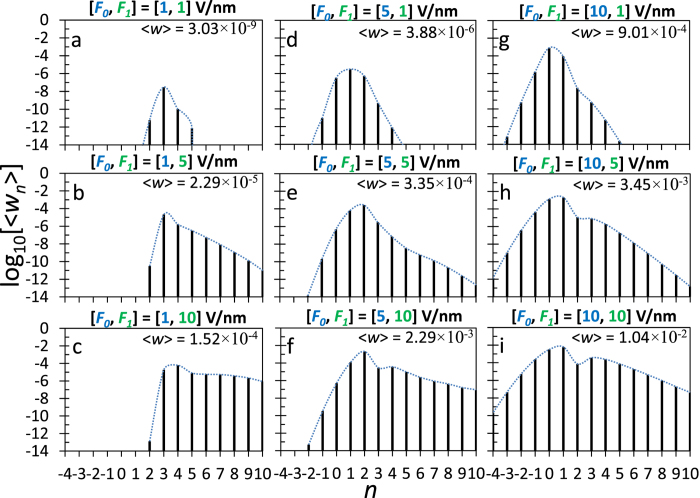
Time-averaged normalized emission current density 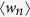 through the *n*th channel, under various combination of dc electric field *F*_*0*_ and laser field *F*_*1*_, calculated from eq. (9). The laser wavelength is 800 nm (corresponding to 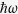 = 1.55 eV). The metal is assumed to be gold, with *E*_*F*_ = 5.53 eV and *W* = 5.1 eV. The dashed lines represent the envelopes of 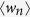. For the laser electric field *F*_*1*_ = 1, 5, and 10 V/nm, the corresponding laser intensity *I* [W/cm^2^] = *ε*_0_*cF*_1_^2^/2 = 1.33 × 10^11^ × (*F*_*1*_[V/nm])^2^ is 1.33 × 10^11^, 3.33 × 10^12^, and 1.33 × 10^13^ W/cm^2^, respectively.

Figure 2 shows the time-averaged normalized emission current density 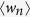 through the *n*th channel, under various combination of dc electric field *F*_*0*_ and laser field *F*_*1*_, calculated from eq. (9). The laser wavelength was chosen to be 800 nm, or a photon energy of 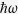 = 1.55 eV. The metal is assumed to be gold, with Fermi energy *E*_*F*_ = 5.53 eV and the work function *W* = 5.1 eV. Unless stated otherwise, these would be the default values for the calculations in this paper. It is clear that the total emission current density 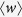 increases when either *F*_*0*_ or *F*_*1*_ increases. When both *F*_*0*_ and *F*_*1*_ are small (Fig. 2a), the dominant emission process is the three-photon absorption, which is consistent with the ratio of the work function to the photon energy 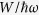 = 3.29, where the electrons at the Fermi level need to absorb at least three photons to substantially reduce the effective potential barrier *W* (Fig. 1a). As *F*_*1*_ increases, higher order channels become important (Fig. 2b,c). In Fig. 2c, all the emission processes with 3 ≤ *n* < 10 contribute significantly to the emitted current. The energy distribution of emission current (i.e. the envelope in Fig. 2a–c), exhibiting a transition from narrow-peaked spectrum to a broad plateau-like spectrum, shows striking resemblance to the experimentally measured kinetic energy spectra^17^, for the transition from multiphoton regime to the strong field regime (c.f. the measured spectra in the insets of Fig. 2a in ref. 17). In Fig. 2c, the emission process with highest probability shifts to four-photon absorption, this is consistent with the well-known feature of channel closing when considering the laser field only (i.e. *F*_*0*_ = 0)^28^,^29^. The minimum value of *n* increases if the given laser field *F*_*1*_ increases, as determined by the drift kinetic energy of the electrons 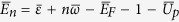, which has to be nonnegative. However, complete channel closing is not observed, especially for cases with larger dc electric field *F*_*0*_ (Fig. 2d–i), where substantially more channels are opened for electron emission. The number of possible emission channels increases with both *F*_*0*_ and *F*_*1*_. Besides multiphoton absorption (*n* > 0), electrons may also emit through processes such as optical direct tunneling (*n* = 0) and multiphoton emission (*n* < 0). As the dc field increases, the effective potential barrier becomes narrower (Fig. 1a), which would not only increase the probability for electron emission through existing channels, but also facilitate electron emission through more channels. For a given *F*_*0*_, increasing *F*_*1*_ tends to shift the dominant emission process to channels with larger *n*, which is consistent with the trend shown in the experiment by Schenk *et al.*^37^. In particular, Schenk *et al.*^37^ observed the electron emission yield at the *S = *1 peak exceeds that of *S = *0 peak at a laser intensity of ~2 × 10^11^ W/cm^2^ for the illumination of a sharp tungsten tip, where *S = n* − 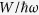 is the above-threshold order. Similar peak shift is noted in Fig. 2b–c, and in Fig. 2d–e. For a given *F*_*1*_, increasing *F*_*0*_ tends to bring the dominant emission process closer to optical tunneling *n* = 0.

**Figure 3:**
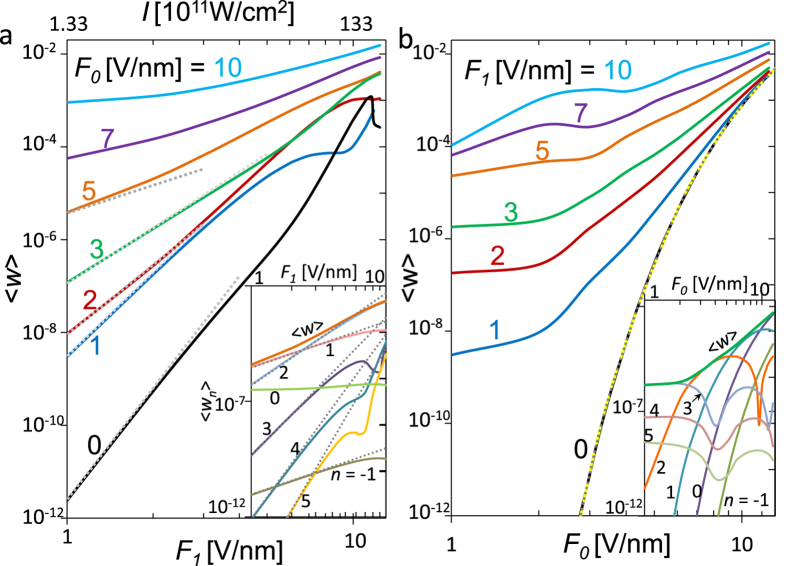
The time-averaged normalized total current density 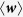 calculated from eq. (9). (**a**) 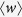 as a function of laser electric field *F*_*1*_ (and laser intensity *I*), for various dc electric fields *F*_*0*_. The special case for *F*_*0*_ = 0 is calculated from eq. (11). The dotted lines represent the scale 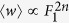, with *n* = 1, 2, 2.5, 3 and 4 (top to bottom, corresponding to F_0 = 5, 3, 2, 1, 0 V/nm, respectively). The inset shows the contribution from individual *n*th channels for the specific case of *F*_*0*_ = 5 V/nm, with the dotted lines for 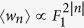. (**b**) 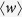 as a function of dc electric fields *F*_*0*_ for various laser electric fields *F*_*1*_. The dashed line is for eq. (12), and the dotted line is for the FN equation. The inset shows the contribution from individual *n*th channels for the specific case of *F*_*1*_ = 3 V/nm. The laser intensity is related to the laser electric field as *I* [W/cm^2^] = 1.33 × 10^11^ × (*F*_*1*_[V/nm])^2^.

Figure 3a shows the time-averaged normalized total emission current density 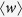 as a function of laser electric field *F*_*1*_ (and laser intensity *I*) for various dc electric fields *F*_*0*_. When *F*_*1*_ < 2 V/nm (*I* < 5.32 × 10^11^ W/cm^2^), the emission current density is well scaled as 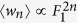, indicating the dominant *n*-photon process. The value of *n* decreases as the dc electric fields *F*_*0*_ increases, which is consistent with the results in Fig. 2.

For the special case of zero dc electric field *F*_*0*_ = 0, the solution in the previous section is simplified by replacing eq. (4) with 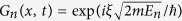, so that *T*_*n*_ is still calculated from eq. (6), with eq. (7a) unchanged, but eq. (7b) replaced by 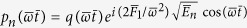 and 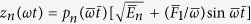. The normalized emission current density in eqs (8) and (9) is modified to read,


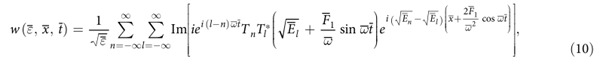



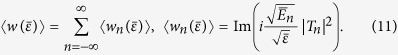


Equation (11) is identical to eq. (46) of Yalunin *et al.*^20^. The emission current density for *F*_*0*_ = 0 calculated from eq. (11) is also shown in Fig. 3a, which serves as the lower limit for *F*_*0*_ → 0. When *F*_*0*_ = 0, four-photon absorption is the dominant emission process, as 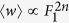 with *n* = 4. The abrupt drop of 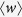 around *F*_*1*_ = 11.6 V/nm is due to the channel closing effect^20^, which is accurately predicted by letting 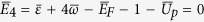, giving *F*_*1*_ = 11.74 V/nm.

When *F*_*0*_ is non zero but small (1 V/nm), the sharp peak of 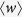 near the channel closing region is smeared, however, there is still a clear transition from multiphoton absorption to optical tunneling emission, indicated by a slope change around *F*_*1*_ ~ 10 V/nm (*I *~ 1.33 × 10^13^ W/cm^2^). The corresponding Keldysh parameter, which is usually used to characterize this transition, is 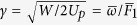 ≈ 1.8. For *F*_*1*_ < 10 V/nm, the emission current is well scaled as 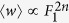 with *n* = 3, indicating the dominant three-photon absorption. For *F*_*1*_ > 10 V/nm, the scale 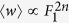 no longer applies, as the emission enters the optical field emission regime.

When *F*_*0*_ becomes large (≥2 V/nm), the slope of 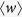 changes gradually as *F*_*1*_ increases, indicating that multiple processes of high photon numbers contribute, and that the dominant process changes as *F*_*1*_ increases, consistent with Fig. 2. The insets in Fig. 3a shows the contribution of different processes for the case *F*_*0*_ = 5 V/nm. The dominant process changes from single-photon absorption (*n* = 1) when *F*_*1*_ < 2 V/nm (*I* < 5.32 × 10^11^ W/cm^2^) to two-photon absorption (*n* = 2) when *F*_*1*_ > 2 V/nm (*I* > 5.32 × 10^11^ W/cm^2^). The individual *n*-photon absorption follows closely the scaling 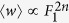 for *n* ≥ 1, when *F*_*1*_ is small. The small dips along *F*_*1*_ are due to the channel closing effects^20^,^28^. The direct tunneling process (*n* = 0) is almost independent of *F*_*1*_. The single-photon emission process (*n* = −1) follows the same scaling as the single-photon absorption (*n* = 1), with 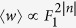, but with a substantially smaller emission current density.

Figure 3b shows the total time-averaged emission current density 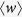 as a function of dc electric fields *F*_*0*_ for various laser electric fields *F*_*1*_. When *F*_*0*_ is small (<2 V/nm), 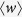 is insensitive to *F*_*0*_ for small *F*_*1*_ (≤5 V/nm), because the dominant emission mechanism in this regime is multiphoton absorption, which presumably is independent of the dc field *F*_*0*_. For larger *F*_*1*_ (≥7 V/nm), 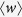 increases with *F*_*0*_, since optical field emission would be important and applying a larger *F*_*0*_ will further assist the tunneling by lowering the potential barrier. When *F*_*0*_ is large (>3 V/nm), Fowler-Nordheim like field emission^20^ from the dc electric field becomes important, thus 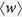 increases with *F*_*0*_, for all the values of *F*_*1*_. As *F*_*0*_ further increases (>8 V/nm), 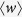 approaches that of field emission in static electric field (i.e. *F*_*1*_ = 0), which, when calculated from eq. (9), gives identical emission current density to that of the known exact solution^31^,^32^,


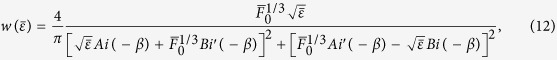


with 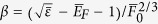. This may serve as one validation of our analytical solution. Note that eq. (12) reduces to the Fowler-Nordheim (FN) equation, 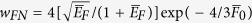, in the limit of 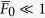, which is also plotted in Fig. 3b. In the regime of large *F*_*0*_, a larger *F*_*1*_ increases <w> even further. Note that the trends of 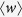 in Fig. 3b are very similar to those in the voltage- and laser power-dependent electron flux measured experimentally by Ropers *et al.* (c.f. Fig. 2c of ref. 2). The insets in Fig. 3b shows the contribution of different emission processes for the case *F*_*1*_ = 3 V/nm. The dips for the individual *n*-photon absorption is due to the channel closing effects^20^,^28^. As *F*_*0*_ increases, the dominant emission process changes continuously, from three-photon absorption (*F*_*0*_ < 2 V/nm), to two-photon absorption (2 V/nm <*F*_*0*_ < 5 V/nm), to single-photon absorption (5V/nm < *F*_*0*_ < 10 V/nm), and eventually to direct tunneling with *n* = 0 (*F*_*0*_ > 10 V/nm).

**Figure 4:**
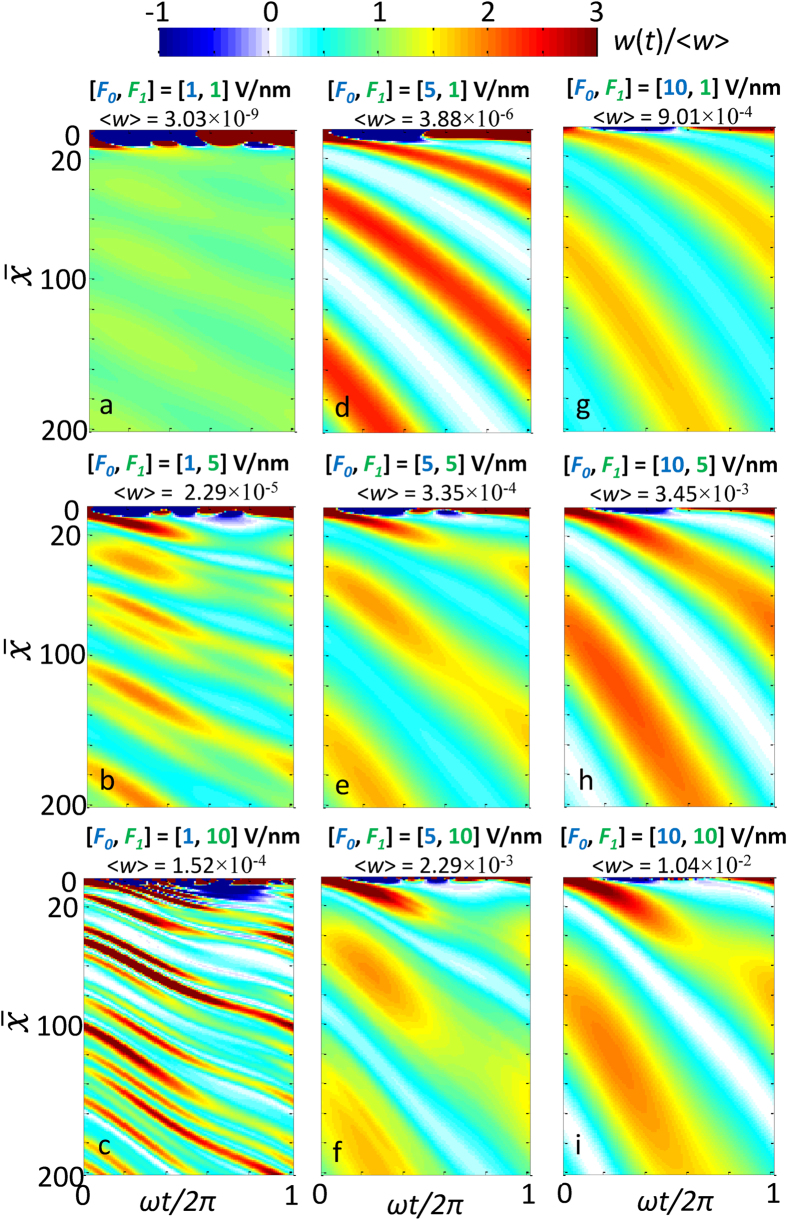
Time-dependent total emission current density *w*(*x*, *t*) normalized to the time-average emission current density 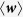, as a function of time and space, under various combination of dc electric field *F*_*0*_ and laser field *F*_*1*_, calculated from eq. (8). For the laser electric field *F*_*1*_ = 1, 5 and 10 V/nm, the corresponding laser intensity *I* = *ε*_0_*cF*_1_^2^/2 is 1.33 × 10^11^, 3.33 × 10^12^ and 1.33 × 10^13^ W/cm^2^, respectively.

**Figure 5:**
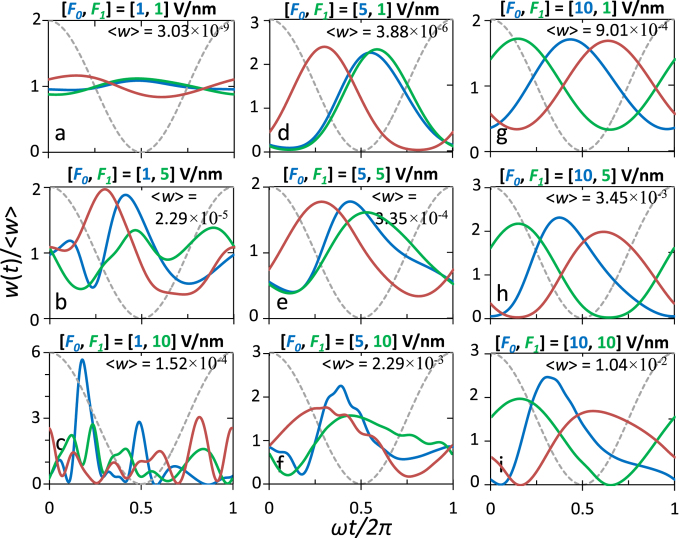
Sample curves in Fig. 4 at 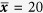 (blue), 100 (green), and 200 (red). Solid lines are the emission current density (or emission rates), and dashed lines indicate the laser field. For the laser electric field *F*_*1*_ = 1, 5, and 10 V/nm, the corresponding laser intensity *I* = *ε*_0_*cF*_1_^2^/2 is 1.33 × 10^11^, 3.33 × 10^12^ and 1.33 × 10^13^ W/cm^2^, respectively.

Figures 4 and 5 show the time-dependent total electron emission current density *w*(*t*), under various combination of dc electric field *F*_*0*_ and laser field *F*_*1*_, calculated from eq. (8). The average emission current is the same as those shown in Fig. 2. The emission current is periodic in time, producing electron bunches with the same period as the laser field 2*π*/*ω*. This is consistent with the experimental observation of periodic variation (at the laser period) of the electron yield due to the carrier-envelope phase (CEP) effects^17^. It is interesting to note from eq. (8) that the emission current from each individual channel, 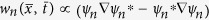 (i.e. set *n* = *l* in eq. (8)) has only a 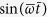 dependence in time for large 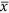, thus is always *π*/2 out of phase with the laser field 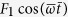. However, this is not the case for the total emission current, as shown in Figs 4 and 5. The complicated shape and phase shift of the total emission current are due to the highly *nonlinear* cross-terms between different channels (i.e. terms with *n* ≠ *l* in eq. (8)). Note that the instantaneous emission probability does not necessarily go to zero when the net electric field is pointing away from the surface, because of highly nonlinear dynamics of electrons near the metal-vacuum interface. This nonlinear process has been thoroughly discussed in previous work (e.g. Figs 5 and 6 of ref. 20, or Fig. 5 of ref. 38). In general, increasing the laser field *F*_*1*_ would increase the peak-to-average ratio of the emitted current, meanwhile reducing the FWHM of the current pulse. In contrast, increasing *F*_*0*_ tends to increase the FWHM of the current pulse. Most importantly, for a given laser field *F*_*1*_, increasing the dc electric field could substantially lower the valley of the current pulse, sometimes even close to zero (Figs 4d,h,i and 5d,h,i), thus giving an intensive modulation of the emitted current. When the dc bias *F*_*0*_ is sufficiently high, profound modulation of the current emission is even possible for a relative small *F*_*1*_, (Figs 4d,g and 5d,g), i.e. for a low intensity laser. It is interesting to note that, at locations 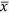 larger than 10 (beyond the localized surface current oscillations, as shown in Fig. 4), the current keeps approximately a fixed temporal profile with only a phase shift, especially when dc bias *F*_*0*_ is larger than laser field *F*_*1*_ (Figs 5a,d,g,h). Physically, the phase shift is primarily due to the drift and acceleration motion of the electrons under the influence of dc and laser fields. The time dependent emission current and its relative phase shift would be important for the study of carrier envelope phases (CEPs)^1^,^17^,^19^,^39^.

**Figure 6:**
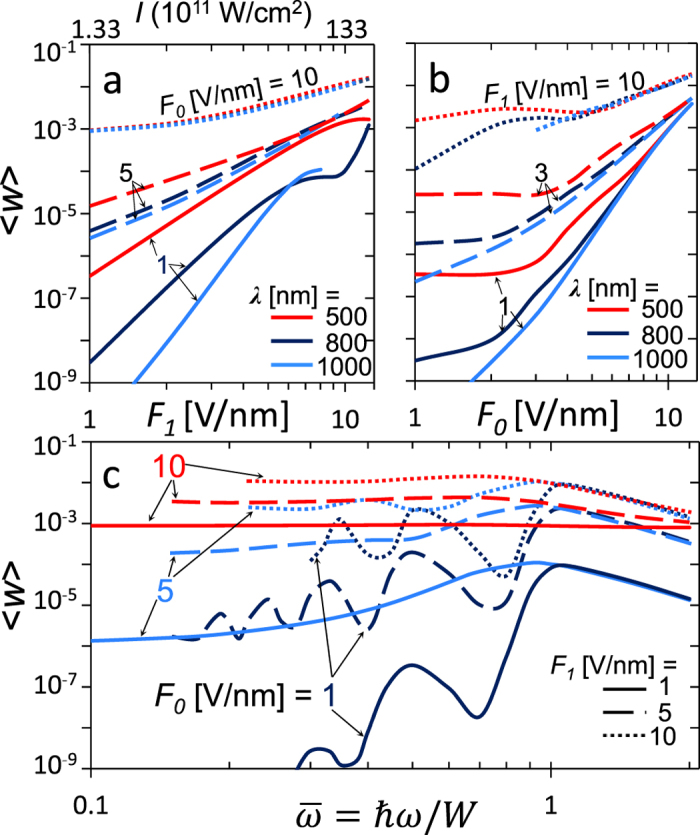
The effects of laser frequency (or wavelength) on the electron emission current density 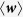. (**a**) 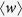 as a function of *F*_*1*_ (and *I*), for various wavelength *λ* with different *F*_*0*_. (**b**) 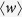 as a function of *F*_*0*_, for various *λ* with different *F*_*1*_. (**c**) 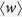 as a function of laser frequency 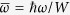, for various combinations of *F*_*1*_ and *F*_*0*_. For the laser electric field *F*_*1*_ = 1, 5 and 10 V/nm, the corresponding laser intensity *I* [W/cm^2^] = 1.33 × 10^11^ × (*F*_*1*_[V/nm])^2^ is 1.33 × 10^11^, 3.33 × 10^12^ and 1.33 × 10^13^ W/cm^2^, respectively.

Figure 6 shows the effects of laser frequency (or wavelength) on the emission current density. When the dc electric field *F*_*0*_ is small, the effects of laser frequency become more pronounced. Figure 6a plots the current emission 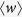 as a function of laser field *F*_*1*_ (and laser intensity *I*), for various combinations of laser wavelength and *F*_*0*_. When *F*_*0*_ = 1 V/nm, the emitted current follows closely the power scale 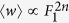 in the multiphoton regime when *F*_*1*_ is small, where *n* is determined by the integer round off of the ratio 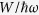 = 2.06, 3.29, and 4.11, for wavelength of 500 nm, 800 nm and 1000 nm, respectively. The laser field *F*_*1*_ for the transition from multiphoton absorption to optical field emission increases with the laser frequency, consistent with the Keldysh parameter, 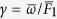. For larger *F*_*0*_, the effects of laser frequency become less important. When *F*_*0*_ = 10 V/nm, the electron emission depends little on the laser frequency. Figure 6b confirms that the effects of laser frequency are important only when the dc bias is small. Figure 6c plots the current emission 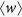 as a function of laser frequency, for various combinations of *F*_*0*_ and *F*_*1*_. When *F*_*0*_ = 1 V/nm, there are distinct peaks near 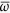 = 1/*n* (*n* > 1), corresponding to the *n*-photon process, with decreasing electron emission current as *n* increases. For 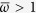, the energy of single photon exceeds the potential barrier *W*, thus the electron emission process is similar to that of over-barrier ionization, where the emission current density decreases as frequency increases. When *F*_*0*_ = 5 V/nm, the *n*-photon peaks are smeared for 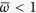; however, the emission current densities for 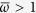
approach those of *F*_*0*_ = 1 V/nm, indicating the over-barrier absorption. When *F*_*0*_ = 10 V/nm, the emission current density becomes almost independent of the laser frequency for 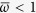, since the dominant emission process is the Fowler-Nordheim like field emission. When 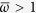, both dc field emission and over-barrier emission would be important, where the dominant process is determined by the strength of the laser field *F*_*1*_. It is important to note that the maximum emission current density is always around 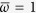.

**Figure 7:**
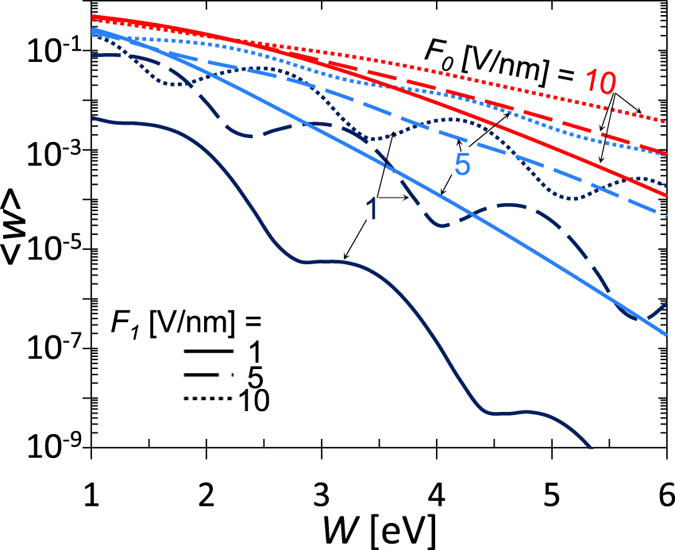
Effects of the barrier height (or work function) W on the emission current density 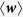. For the laser electric field *F*_*1*_ = 1, 5, and 10 V/nm, the corresponding laser intensity *I* [W/cm^2^] = 1.33 × 10^11^ × (*F*_*1*_[V/nm])^2^ is 1.33 × 10^11^, 3.33 × 10^12^, and 1.33 × 10^13^ W/cm^2^, respectively.

Figure 7 shows the effects of the barrier height (or work function) *W* on the emission current density, for *E*_*F*_ = 5.53 eV and *ħω* = 1.55 eV (*λ* = 800 nm). In general, the emission current density increases as the work function decreases. When both *F*_*0*_ and *F*_*1*_ are small, there are peaks around *W*/*ħω* = *n*, corresponding to the resonant *n*-photon absorption. Similar results are obtained by previous numerical calculation^24^. When *F*_*1*_ increases, the oscillating features persist, however, the location of the peaks shifts, due to both channel closing effects as well as the optical field tunneling process. Increasing dc bias *F*_*0*_ would reduce the transmission peaks. One may notice that the emission current density increases even when *W* < *ħω*, whereas in Fig. 6c, the emission current density decreases when *ħω* /*W* > 1. The discrepancy arises because the relative value of the Fermi level *E*_*F*_/*W* (thus the normalized electron initial energy) changes when *W* changes in Fig. 7, but remains the same when the laser frequency changes in Fig. 6c.

**Figure 8:**
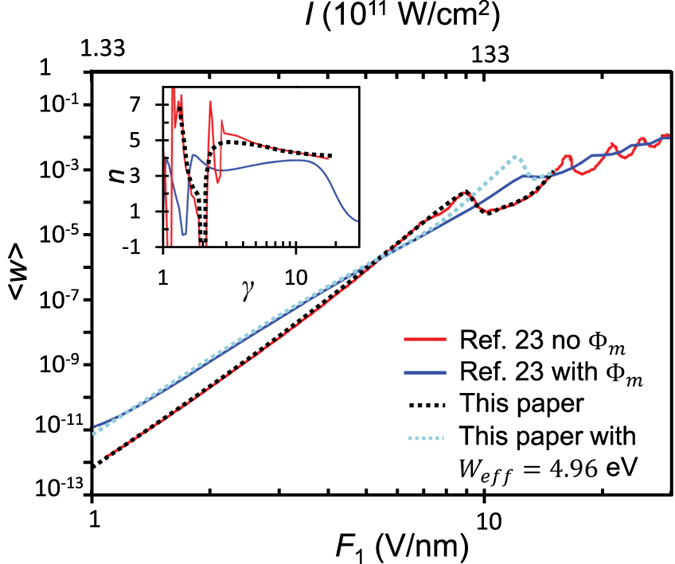
Comparison of our exact solution in this paper, eq. (9) with the numerical simulation in ref 23, for two cases with and without image charge potential Φ_m_. The inset shows the multiphoton order *n* as a function of Keldysh parameter *γ*. The laser intensity is related to the laser electric field as *I* [W/cm^2^] = 1.33 × 10^11^ × (*F*_*1*_[V/nm])^2^.

It is clear that our exact analytical solution is derived based on the simple potential landscape defined in eq. (1). It is important to know the sensitivity of such calculations to the shape of the surface potential. The effects of more realistic surface potential have been studied previously, by solving directly the time-dependent Shrodinger Equation (TDSE) numerically, for example in refs 1 and 23. In Fig. 8, we compare our analytical calculation using potential landscape eq. (1) with the numerical simulations in ref. 23, which is for the more realistic potential landscape outside the metal surface (*x* > 0), Φ (*x*,*t*) = Φ_m_(*x*) + *V*_0_ − *eF*_0_*x* − *exF*_1_exp(−2ln2*t*^2^/*τ*^2^)cos*ωt*, where Φ_m_(*x*) = −*e*^2^/(16*πε*_0_*x*) is the image charge potential, the last term represents a finite Gaussian laser pulse of pulse length *τ*, and the remaining parameters are defined in eq. (1). In Fig. 8, the following parameters are used^23^, work function *W* = 5.5 eV, Fermi energy *E*_*F*_ = 4.5 eV, laser wavelength is 800 nm, *τ* = 30 fs (~11 laser cycles), and dc electric field *F*_*0*_ = 0.2 V/nm. For the potential profile without Φ_m_, our analytical results for the emission current density 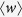 vs. laser field *F*_*1*_ are in excellent agreement with the numerical simulations. The inset in Fig. 8 shows the multiphoton order *n* as a function of Keldysh parameter *γ*, by fitting the slope of 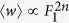, again, showing very good agreement with the numerical simulation. These results serve yet another validation of our exact solution. More importantly, it indicates that our exact solution using infinite plane wave laser excitation is even applicable to more realistic laser profiles, as long as the pulse length is larger than the wavelength. With the inclusion of Φ_m_, the slope of the multiphoton regime is reduced in general, due to the reduction of effective potential barrier near the metal surface. It is easy to estimate that the potential maximum is 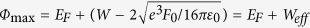 when considering the static potential barrier only (i.e. no laser field), where *W*_eff_ is the effective work function, which is found to be 4.96 eV for the given parameters. Similar approach for the effective work function has been adopted to study above-threshold photoemission^37^. By adopting this effective work function in our analytical calculation, we found fairly good agreement with the numerical simulation up to *F*_*1*_ ~ 8 V/nm. Thus, by replacing *W* with *W*_eff_, our solution may still be used to estimate the electron emission characteristics in the multiphoton regime, even for potential profiles with image charge.

## Summary

In summary, we have presented an exact solution to laser induced strong-field electron emission from a metal surface under dc bias. We analytically solve the time-dependent Schrödinger equation with both a single frequency oscillating field *F*_*1*_cos*ωt* and a static electric field *F*_*0*_. The solution takes the general form of the superposition of electron plane waves with ladder-state energies (channels) separated by the photon energy 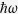 coupled to the electron’s initial energy. It is found that increasing the dc bias *F*_*0*_ would increase the electron emission, by opening up more channels, including the processes such as multiphoton absorption, optical tunneling, multiphoton emission, and field emission. When *F*_*0*_ is small, clear transition from the multiphoton absorption regime to the optical field emission regime is shown. When *F*_*0*_ is large, multiple processes contribute to the electron emission. In the limit of *F*_*0*_ → 0, the solution approaches that of Yalunin *et al.*^20^, for the special case of *F*_*0*_ = 0. In the limit of large *F*_*0*_, the solution recovers the Fowler-Nordhiem dc field emission. The time-dependent emission current reveals that intense current modulation may be possible even with a low intensity laser, by merely increasing the applied dc bias. The effects of laser frequency and metal work function are also examined in detail. It is found that for given values of electric fields, *F*_*0*_ and *F*_*1*_, the maximum electron emission current density always occurs around the laser frequency *ħω*/*W* = 1. The emission current decreases with the laser frequency when *ħω*/*W* > 1. All electron emission mechanisms are captured in a single formulation, demonstrating transition from *n*-photon process to optical and field tunneling.

Our theoretical results exhibit good coincidence with previous experimental measurements on: measured kinetic energy spectra of the photoelectrons, for the transition from multiphoton regime to the strong field regime^17^; shifts on peak of energy spectra^28^,^29^,^37^; the scaling of voltage- and laser power-dependent measured electron flux^2^; and periodic variation (at the laser period) of the electron yield due to the carrier-envelope phase (CEP) effects^17^. Thus, our exact analytical solution provides an efficient tool to analyze the complicated nonlinear dynamics of laser induced electron emission. It offers plausible interpretation of experimental results, and guidance of future experimental design.

While our results are derived for continuous-wave excitation, they are shown to provide a good approximation to time-varying or pulsed fields (Fig. 8), as long as the optical frequency is much larger than the bandwidth of the driving field^4^,^20^. A slowly varying envelope approximation, then allows a straightforward extension of our results to pulsed excitation, as stressed in ref. 20.

In this formulation, we have made the following assumptions: 1) the electrons are emitted from a single energy level at the Fermi level; 2) the surfaces of the electrodes are flat and the problem is assumed one-dimensional; 3) the image charge potential is not directly included to make the analytical treatment possible. The effects of electrode geometry^40^,^41^, space charge^6^,^36^,^42^,^43^, nature of the ion lattice of the electrodes, thermal redistribution, and short pulse illumination may be considered in future studies.

## Methods

Following Truscott^33^, the time dependent potential for x ≥ 0 may be written as 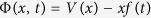, with 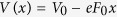, and 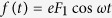. Thus, eq. (2) may be transformed to the coordinate system *ξ*, *t*, where 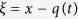, the displacement 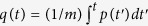, and 
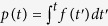
, by assuming that 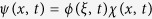, with 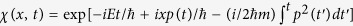. We have,


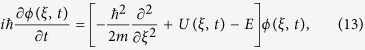


with 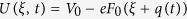. By separation of variables, eq. (13) can be easily solved to give


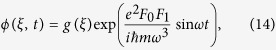


where 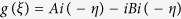 is the solution of the “static” equation^31^,^32^ 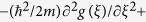

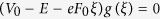
, where 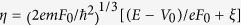. From 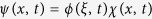, we obtain eq. (3), which is the exact solution to eq. (2), upon using 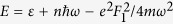.

## Additional Information

**How to cite this article**: Zhang, P. and Lau, Y. Y. Ultrafast strong-field photoelectron emission from biased metal surfaces: exact solution to time-dependent Schrödinger Equation. *Sci. Rep.* **6**, 19894; doi: 10.1038/srep19894 (2016).
